# Epidemiological shifts in chronic kidney disease: a 30-year global and regional assessment

**DOI:** 10.1186/s12889-024-21065-9

**Published:** 2024-12-18

**Authors:** Kaili Qin, Jianbo Qing, Qian Wang, Yafeng Li

**Affiliations:** 1 The Fifth Clinical Medical College, Shanxi Medical University, Taiyuan, 030001 China; 2 The Third Clinical Medical College, Zhejiang University School of Medicine, Hangzhou, 310000 China; 3 Department of Nephrology, Sir Run Run Shaw Hospital, Zhejiang University, Hangzhou, 310000 China; 4 Shanxi Provincial Key Laboratory of Kidney Disease, Taiyuan, Shanxi China; 5 Department of Nephrology, Shanxi Provincial People’s Hospital (Fifth Hospital), Shanxi Medical University, Taiyuan, 030001 China; 6 Chronic Kidney Disease Medical and Pharmaceutical Basic Research Innovation Center of the Ministry of Education of the People’s Republic of China, Taiyuan, China; 7 Core Laboratory, Shanxi Provincial People’s Hospital (Fifth Hospital), Shanxi Medical University, Taiyuan, 030001 China; 8 Academy of Microbial Ecology, Shanxi Medical University, Taiyuan, 030001 China; 9 Hejin municipal People’s Hospital, Hejin, 043300 China; 10 Department of Nephrology, Shanxi Provincial People’s Hospital (Fifth Hospital) of Shanxi Medical University, Taiyuan, Shanxi 030001 China

**Keywords:** Chronic kidney disease, Epidemiology, Global health, Incidence, Prevalence

## Abstract

**Background:**

Chronic kidney disease (CKD) presents a growing global health challenge, with significant variability in disease burden across different regions and countries. This study aimed to analyze the trends in incidence, prevalence, mortality, and disability-adjusted life years (DALYs) for CKD from 1990 to 2019, utilizing data from the Global Burden of Disease Study.

**Methods:**

We conducted an in-depth study on the global and age-standardized incidence, prevalence, mortality, and DALYs of CKD, and assessed trends over a 30-year period. Additionally, we explored the associations between healthcare access and quality (HAQ), the Socio-Demographic Index (SDI), and CKD. Furthermore, we conducted a detailed analysis of six risk factors closely related to CKD, and based on these findings, provided strong evidence for enhancing the management of CKD.

**Results:**

In 2019, there were 18,986,903 cases of CKD, with an average annual percent change (AAPC) of 1.82 (95% CI = 1.8 to 1.82) in incidence since 1990. The age-standardized incidence rate increased from 192.45 per 100,000 in 1990 to 233.65 per 100,000 in 2019. Prevalence also rose, with a total of 69,729,430 cases in 2019 and an AAPC of 1.19 (95% CI = 1.19 to 1.2). Mortality and DALYs have increased correspondingly, with the mortality rate reaching 18.29 per 100,000 and total DALYs at 41,538,592 in 2019. The analysis showed that higher HAQ levels are associated with better outcomes in terms of lower mortality and DALY rates, whereas lower HAQ levels correlate with poorer outcomes. In addition, high fasting plasma glucose and high systolic blood pressure are the main contributors to CKD-related deaths, with their population attributable fraction (PAF) significantly decreasing as the SDI decreases.

**Conclusion:**

The burden of CKD has significantly increased over the past three decades, influenced by demographic changes and variations in healthcare quality and access. Effective public health strategies and improvements in healthcare delivery are needed to address the disparities in CKD outcomes globally.

**Supplementary Information:**

The online version contains supplementary material available at 10.1186/s12889-024-21065-9.

## Background

Chronic kidney disease (CKD) is defined by either the presence of kidney damage or a reduced glomerular filtration rate (GFR < 60 ml/min/1.73 m^2^) persisting for three months or more [1]. Based on the definition of CKD using GFR, it is categorized into five stages: G1 (≥ 90 ml/min per 1.73 m²), G2 (60–89 ml/min per 1.73 m²), G3a (45–59 ml/min per 1.73 m²), G3b (30–44 ml/min per 1.73 m²), G4 (15–29 ml/min per 1.73 m²), and G5 (< 15 ml/min per 1.73 m²) [2]. This condition progressively and irreversibly alters renal function and structure over months or years [3–5]. CKD significantly increases the risk of premature mortality, which is 5 to 10 times higher than the likelihood of progressing to end-stage renal disease (ESRD). In 2017, nearly 700 million people globally were affected by CKD [6], a prevalence surpassing that of diabetes, osteoarthritis, and chronic obstructive pulmonary disease (COPD) [7]. During that same year, CKD was also responsible for approximately 1.2 million deaths worldwide, surpassing deaths from tuberculosis or HIV and approaching those from road injuries [6]. Extensive research has identified diabetes and hypertension as major contributors for CKD across varying income levels [8–11]. CKD is characterized by the progressive and irreversible depletion of nephron units, diminished renal regenerative capabilities, microvascular damage, metabolic alterations, oxidative stress, and inflammation [5]. This cascade ultimately culminates in fibrosis and the further loss of nephrons. The underlying mechanisms of CKD encompass multiple pathways, notably nephron loss, nephron hypertrophy, compromised glomerular filtration, and fibrosis. Furthermore, a multitude of factors can precipitate the onset of chronic kidney disease, such as low birth weight, pregnancy complications, obesity, diabetes, and advancing age. These conditions not only contribute to but also exacerbate the loss of nephron units, perpetuating a vicious cycle of injury that eventually leads to ESRD [12].

The forces of epidemiologic transition and demographic growth have significantly shaped the epidemiology of non-communicable diseases, including diabetes and hypertension, thus impacting the global landscape of CKD [13]. Despite this, comprehensive analyses of the global, regional, and national CKD burden over the last three decades are scarce.

This study utilizes data from the Global Burden of Disease (GBD) from 1990 to 2019 to perform a comprehensive analysis of the incidence, prevalence, mortality, and disability-adjusted life years (DALYs) associated with CKD at global, regional, and national levels. We also explore the impact of epidemiological changes and population growth on the CKD burden during this period, while examining the relationship between the Socio-demographic Index (SDI) and the Health Care Access and Quality (HAQ) index across different countries. In addition, this study, through a comprehensive analysis of risk factor exposure levels and their associated disease burdens, offers vital insights for the development and implementation of strategies and policies to prevent and mitigate the future escalation of the CKD burden.

## Materials and methods

### Data sources

The data utilized in this report are derived from GBD 2019, which encompasses epidemiological statistics for 369 diseases across 21 GBD regions and 204 countries and territories spanning the period of 1990–2019 [14–16]. The report categorizes all regions into 21 GBD regions based on epidemiological similarity and geographical proximity, and further groups them into five strata according to their socio-demographic index (SDI; low SDI, low-medium SDI, medium SDI, high-medium SDI, and high SDI) [15, 17]. We obtained the incidence cases and rate (per 100,000 population), prevalence cases and rate (per 100,000 population), mortality cases and rate (per 100,000 population) disability-adjusted life years (DALYs) count and rate (per 100,000 person-year) by sex, age, region and country. The age standardization utilized the World Health Organization’s standard age structure for the world population as its basis [13]. The study adheres to the Guidelines for Accurate and Transparent Health Estimates Reporting (GATHER) [18].

### Socio-demographic Index (SDI)

The SDI is a composite measure of development, ranging from 0 to 1. It is estimated based on three indicators: the overall fertility rate among women under the age of 25 (TFU25), the average educational attainment among adults aged 15 years and older (EDU 15+), and the lagging per capita income distribution (LDI). Zero indicates that the country or region has the highest TFU25 and lowest EDU 15 + and LDI, while an SDI representation of 1 signifies the opposite meaning [19, 20].

### Health care access and quality (HAQ) index

The HAQ index offers a comprehensible metric ranging from the lowest (0) to the highest (100), based on risk-standardized mortality rates for 32 causes of GBD [21]. This facilitates a standardized evaluation of healthcare access and quality across 195 countries and territories, considering temporal and developmental variations.

### Risk factors

The risk factors for CKD encompass high systolic blood pressure, elevated fasting plasma glucose, high body mass index (BMI), dietary risks, low physical activity, and environmental risks. In the GBD study, these risk factors were carefully selected based on robust causal evidence, the availability of exposure data, potential for modification through behavior or other means, and relevance to health policy. For GBD 2019, risk-outcome pairs that met the World Cancer Research Fund’s criteria for convincing or probable evidence were included in the compilation of relative risk data. The relative risks for these pairs were determined using a comprehensive approach [22, 23]. Exposure data for these risk factors were sourced from various reliable channels: high systolic blood pressure data were drawn from literature and household survey microdata (e.g., STEPS, NHANES); high FPG data were compiled from estimates of mean FPG in representative populations, individual-level survey data, and diabetes prevalence estimates; high BMI was defined for adults (aged 20+) as BMI > 20–25 kg/m², with dietary risk data sourced from PubMed literature searches and updates from the IHME Global Health Data Exchange; low physical activity was assessed by measuring activity lasting at least 10 min across all life domains for adults aged 25+, while environmental risk factors like radon and lead exposure were evaluated using expert-managed datasets, national surveys, government reports, and scientific literature [24]. The population attributable fraction (PAF) was calculated to reflect the impact of these risk factors, indicating the proportion of outcomes that could be eliminated if the risk factor were removed. In GBD 2019, PAFs were determined by considering the risk function, exposure distribution across individuals in each age-sex-location-year, relative risk estimates from meta-analyses, and theoretical minimum risk levels for each risk-outcome pair [25].

### Statistical analysis

In order to investigate the temporal changes in various indicators from 1990 to 2019, the age-standardized rate of estimated annual percentage change (ASR-EAPC) measure was used to assess trends over a specific time period [26–29]. Utilizing data sourced from the Global Burden of Disease (GBD) database, we calculated age-standardized incidence rates (ASIR), age-standardized prevalence rates (ASPR), age-standardized mortality rates (ASMR), and age-standardized disability-adjusted life years rates (ASDR) for CKD at global, regional, and country levels. Additionally, a world map was generated. EAPC (Estimated Annual Percentage Change) represents the estimated annual percentage change calculated using a linear regression model expressed as ln (y)=α + βx + ε, where the dependent variable y can be ASIR, ASPR, ASMR, or ASDR; α is the intercept; x represents the year; β is the slope; and ε is normally distributed. The EAPC is computed as EAPC=(exp^β-1)×100%, from which the 95% confidence intervals (CI) for the EAPC can be derived. A positive EAPC value, along with a lower limit of the Cl greater than zero, indicates an increasing trend in ASR. Conversely, if the upper limit of both EAPC and Cl is less than zero, it suggests a decreasing tendency in ASR. Furthermore, an investigation into different life stages was conducted by analyzing ASIR, ASPR, ASMR, and ASDR with respect to age and gender.

We calculated the average annual percent changes (AAPCs) by using the Joinpoint regression model with logarithm-transformed rates [30–32], as the dependent variable and year as the independent variable. The incidence rate, prevalence rate, mortality rate, and DALYs rate were evaluated at global, regional, and country levels using AAPCs. AAPC values and 95% confidence interval (CI) represents the percentage change (increase, decrease, or no change) observed from year to year. For instance, an AAPC value of 0.5 indicates an annual growth rate of 0.5%. Moreover, the Person correlation coefficient R was employed to ascertain the association between age-standardized rates and SDI across various regions and countries [33]. Additionally, an investigation was conducted to comprehend the distribution of CKD in health systems across different nations by examining the relationship between ASIR, ASPR, ASMR, ASDR, and HAQ [13].

To investigate the impact of aging, epidemiological changes, and population growth on the incidence, prevalence, mortality, and DALYs associated with CKD, we employed the the decomposition methodology of Das Gupta [13, 34–36]. The formula is as follows (e.g. incidence):

$$\:{\text{I}\text{n}\text{c}\text{i}\text{d}\text{e}\text{n}\text{c}\text{e}}_{\text{a}\text{y},\text{p}\text{y},\text{e}\text{y}}={\sum\:}_{\text{i}=1}^{20}\left({\text{a}}_{\text{i},\text{y}}\times\:{\text{p}}_{\text{y}}\times\:\:{\text{e}}_{\text{i},\text{y}}\right)$$

ay, py, and ey presented incidence based on aging, population growth, and incidence for a specific year, respectively. A_i, y_ represents the proportion of the population in y year of a certain i age group among the 20 age groups, p_y_ represents the total population in y year, and e_i, y_ represents the incidence of the ith age group in y year. The contribution of each factor to the change in incidence from 1990 to 2019 was defined as the impact of a change in a factor when other factors were held constant. The R software (version 4.2.2 RFoundation, Vienna, Austria, https://www.r-project.org/) and Joinpoint regression program (version 4.9.1.0, Surveillance Research Program, National Cancer Institute, USA) were utilized for all data analysis purposes in this study. Statistical significance was determined at a two-sided *P*-value < 0.05.

## Results

### The incidence of CKD

**Fig. 1:**
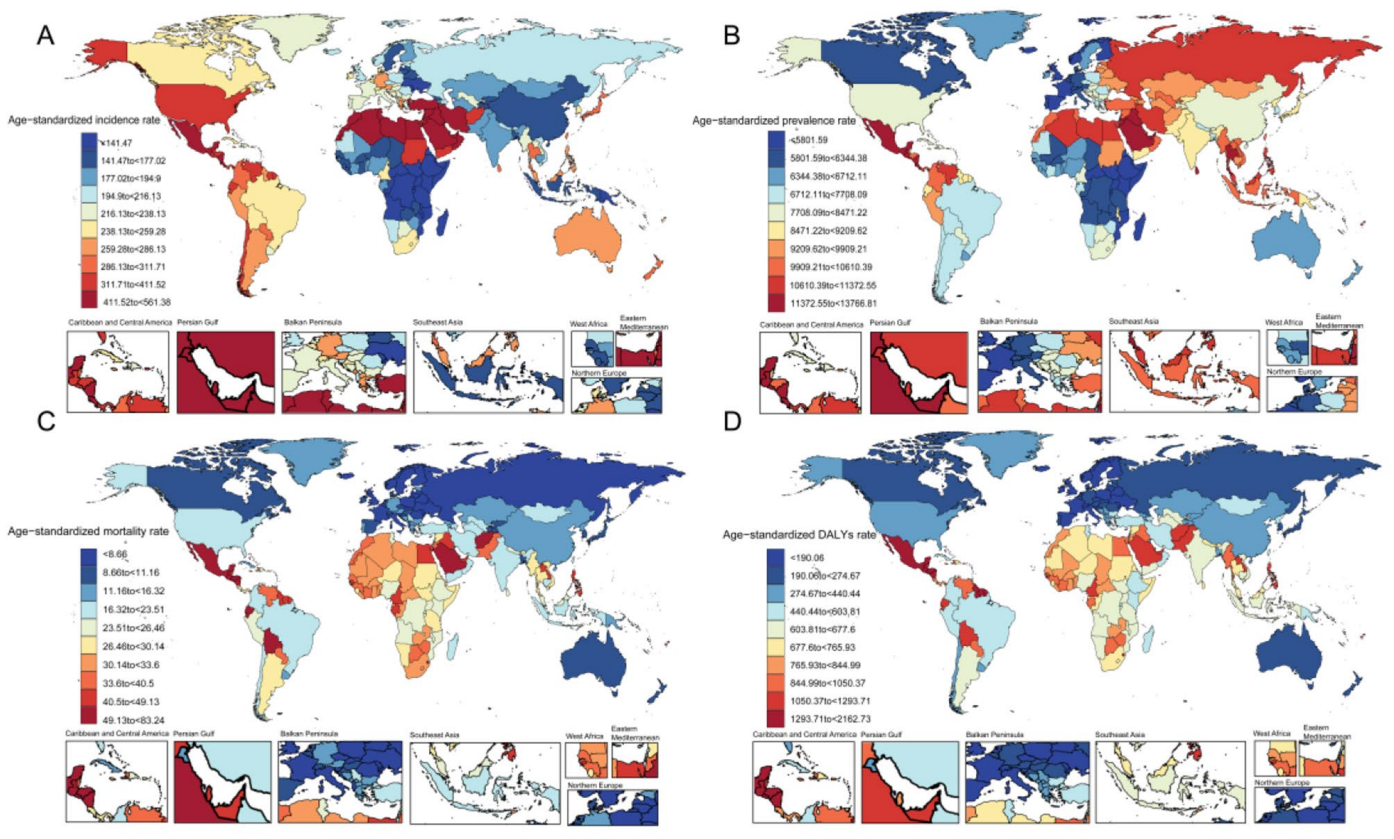
Geographic distribution of age-standardized incidence rate (**A**), age-standardized prevalence rate (**B**), age-standardized mortality rate (**C**), and age-standardized disability-adjusted life years rate (**D**) of CKD in 204 countries and territories

**Fig. 2:**
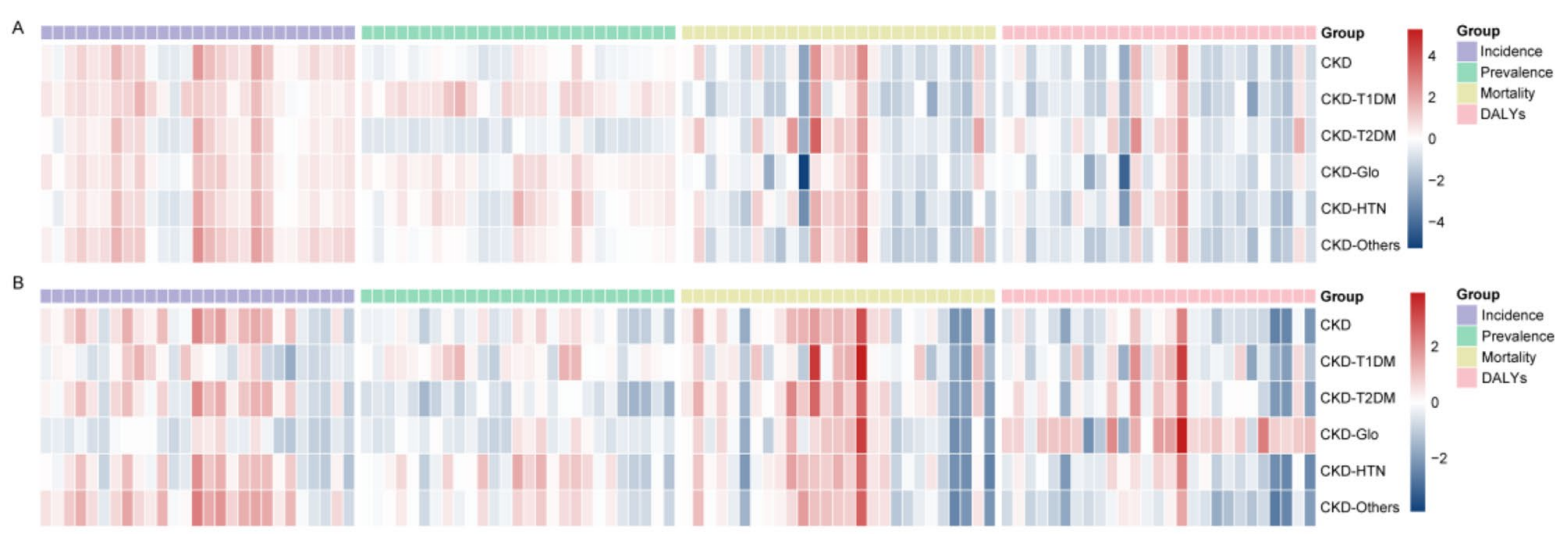
Estimated annual percentage change (**A**) as well as average annual percent change (AAPC) values for the 21 GBD regions (From left to right are Global, High SDI, High-middle SDI, Middle SDI, Low-middle SDI, Low SDI, Central Asia, Central Europe, Eastern Europe, Australasia, High-income Asia Pacific, High-income North America, Western Europe, Andean Latin America, Caribbean, Central Latin America, Southern Latin America, Tropical Latin America, North Africa and Middle East, Southeast Asia, South Asia, East Asia, Oceania, Central Sub-Saharan Africa, Eastern Sub-Saharan Africa, Southern Sub-Saharan Africa and Western Sub-Saharan Africa)

Globally, the number of CKD incidence cases rose from 7,796,328 (95% UI = 7,174,529 to 8,485,391) in 1990 to 18,986,903 (95% UI = 17,556,535 to 20,518,156) in 2019 (Table S1). Over the past three decades, there has been a consistent upward trend in CKD incidence, with an average annual percent change (AAPC) of 1.82 (95% CI = 1.81 to 1.82) from the baseline year of 1990 (Table S1 and Fig. 2). In 2019, the age-standardized incidence rate (ASIR) for CKD was 233.65 per 100,000 population (95% UI = 216.56 to 252.31) (Table S1 and Fig. 1). Additionally, the incidence rate exhibited an increasing pattern over time, with an estimated annual percent change (EAPC) of 0.69 (95% CI = 0.68 to 0.7) (Fig. 2). The incidence of CKD attributed to type 1 diabetes mellitus (T1DM), type 2 diabetes mellitus (T2DM), hypertension, glomerulonephritis, and other unspecified causes also showed an upward trend, compared to data from 1990, with an EAPC of 1.21% (95% CI = 1.08 to 1.34) (Table S1).

According to the quintile of SDI, there was an increasing trend in incidence rates across low, low-medium, medium, high-medium and high SDI areas. The most significant increase was observed in the middle SDI area (AAPC = 2.69; 95% CI = 2.68 to 2.7), while the lowest increase occurred in the low SDI area (AAPC = 1.87; 95% CI = 1.85 to 1.89) (Table S1). At 21 GBD regions level, the ASIR per 100,000 for CKD was found to be highest in North Africa and the Middle East (ASIR = 447.48; 95% UI = 415.13 to 482.83), followed by Central Latin America (ASIR = 409.61; 95% UI = 383.06 to 437.91), and High-income North America (ASIR = 310.44; 95% UI = 284.74 to 336.3) (Table S1 and Fig. 1). Analyzing the incidence trend over the past three decades, a noteworthy surge was observed in Andean Latin America (AAPC = 3.56; 95% CI = 3.55 to 3.57), followed by Central Latin America (AAPC = 3.12; 95% CI = 3.11 to 3.13) and North Africa and Middle East (AAPC = 2.89; 95% CI = 2.87 to 2.9). Importantly, an upward trajectory in incidence was evident across all regions (all AAPC > 0) (Table S1 and Fig. 2).

**Fig. 3:**
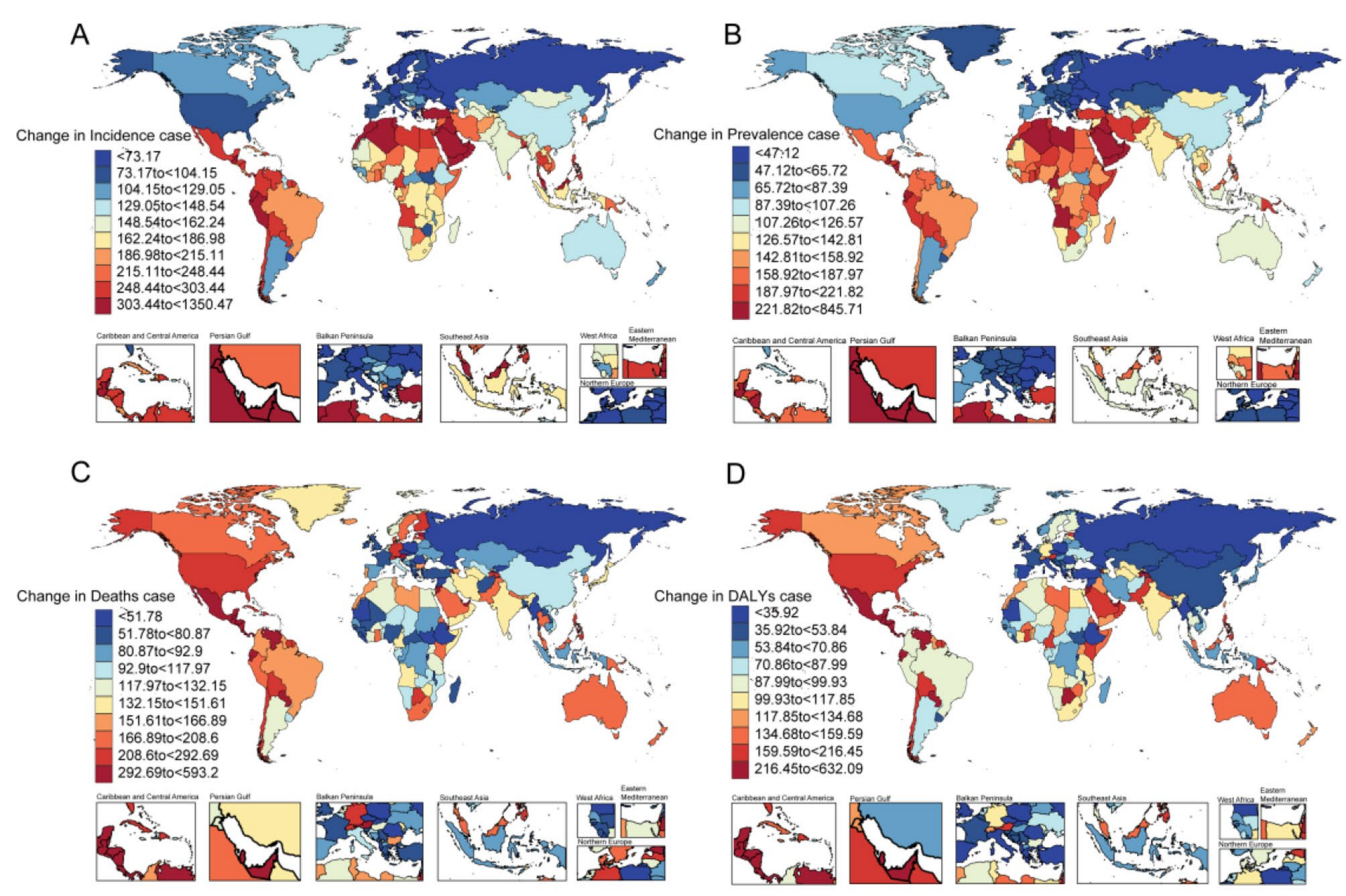
Geographic distribution of change in incidence cases (**A**), change in prevalence cases (**B**), change in deaths cases (**C**), and change in disability-adjusted life years cases (**D**) of CKD in 204 countries and territories

Among the 204 countries and territories, China (3,098,718; 95% UI = 2,812,225 to 3,412,879), India (2,161,590; 95% UI = 1,964,382 to 2,363,305), and United States of America (1,724,082; 95% UI = 1,574,583 to 1,886,709) reported the highest number of cases respectively. Evaluating the trend over time reveals that Albania exhibited the highest growth rate (AAPC = 4.21; 95% CI = 4.14 to 4.27), followed by Bahrain (AAPC = 4.16; 95% CI = 4.12 to 4.20) and Bosnia and Herzegovina (AAPC = 4.15; 95% CI = 4.10 to 4.21). Notably, the magnitude of growth decreased only in Afghanistan (AAPC = -0.24; 95% UI = -0.29 to -0.18), while the rest increased (Table S2). Saudi Arabia had the highest ASIR (561.38 per 100,000 population; 95%UI = 524.55 to 598.58), followed by United Arab Emirates (516.47 per 100,000 population; 95%UI = 476.76 to 558.41) and Qatar (ASIR = 506.35 per 100,000 population; 95%UI = 463.47 to 551.97). The largest increase in ASIR was observed in Morocco (EAPC = 2.65%; 95% CI = 2.57 to 2.72), Turkey (EAPC = 2.46%; 95% CI = 2.27 to 2.66) and Ecuador (EAPC = 2.37%; 95% CI = 2.24 to 2.49). A slight decrease in ASIR was noted among only six out of two hundred four countries or regions (Table S2 and Fig. 2). The rates have witnessed a surge across all countries, with an astounding increase of over 300% in 23 nations since 1990. Notably, Qatar experienced the most substantial growth at an astonishing rate of 1350.47%, closely followed by the United Arab Emirates (1219.43%) (Fig. 3).

### The prevalence of CKD

The global prevalence of CKD has exhibited a significant increase (AAPC = 1.19; 95% CI = 1.19 to 1.2), with a corresponding rise in the number of cases by 104.09% compared to the year 1990. Moreover, there has been an upward trend observed in the ASPR of CKD (EAPC = 0.32%; 95% CI = 0.32 to 0.33) (Table S3). Specifically, the ASPR of CKD escalated from 7855.83 per 100,000 individuals in 1990 to 8596.21 per 100,000 individuals in the year 2019 (Table S3 and Fig. 1). Among the five causes of CKD, the prevalence and ASPR of CKD caused by five causes increased. The ASPR of CKD caused by other and unspecified causes was highest (6342.15; 95% UI = 5905.8 to 6753.79), followed by CKD caused by T2DM (ASPR = 1576.35; 95% UI = 1448.28 to 1700.21). In terms of SDI quintiles, the middle SDI region exhibited the highest number of cases, an increase in incidence, ASPR, and an increase in ASPR among the five regions in 2019 (Table S3).

Among the 21 regions, East Asia exhibited the highest number of GBD cases (156,838,754; 95% UI = 144,507,123 to 169,062,378), while Central Latin America experienced a substantial increase in incidence rates (AAPC = 2.2; 95% CI = 2.20 to 2.21). Notably, Central Latin America recorded the highest ASPR in 2019 (12,139.27; 95% UI = 11,377.05 to 12,843.88). The region of North Africa and Middle East demonstrated the most significant rise in ASPR over time (EAPC = 1.17%; 95% CI = 1.13 to 1.22). Interestingly enough, only one region (High-income Asia Pacific) displayed a declining trend in ASPR (Table S3 and Fig. 2).

In 2019, the countries with the largest number of prevalence cases were China (150,497,490; 95%UI = 138612025 to 162,338,825), India (115,223,088; 95%UI = 106,617,969 to 124,209,571), and United States of America (40,241,611; 95%UI = 37,608,687 to 42,831,133), while three countries with the highest ASPR were Mauritius (13,766.81; 95%UI = 12,855.22 to 14,728.09), Mexico (13,418.53; 95%UI = 12,565.28 to 14,188.77), and Costa Rica (12,859.29; 95%UI = 12,284.46 to 13,498.62) (Table S4). Regarding the increasing trends, the three countries with the most notable increases in prevalence from 1990 to 2019 were Tunisia (AAPC = 2.81; 95% CI = 2.81 to 2.82), Turkey (AAPC = 2.78; 95% CI = 2.76 to 2.79), and Syrian Arab Republic (AAPC = 2.68; 95% CI = 2.65 to 2.71). Among them, only two countries have seen a decrease in prevalence, which are Afghanistan (AAPC = -0.3; 95% CI = -0.33 to -0.27) and Chad (AAPC = -0.16; 95% CI = -0.17 to -0.16). The trend of ASPR has predominantly increased in most countries, with the most significant increase observed in Morocco (EAPC = 1.52%; 95% CI = 1.5 to 1.54). Only six countries have shown a decrease in the trend of ASPR, with the United Kingdom experiencing the most significant decrease (EAPC = -0.09%; 95% CI = -0.14 to -0.04) (Table S4). Between 1990 and 2019, a total of 27 countries witnessed a prevalence surge exceeding 200%, with Qatar exhibiting the most substantial increase (845.71%). Notably, only Georgia demonstrated a decline in prevalence (-2.42%) (Fig. 3).

### The mortality of CKD

The global mortality rate of CKD exhibited a consistent upward trend over the past three decades (AAPC = 1.75; 95% CI = 1.73 to 1.78), with the number of deaths increasing from 601,307 in 1990 to 1,427,232 in 2019. Additionally, there was a slight increase in age-standardized mortality rates (ASMR), rising from 16.14 per 100,000 population to 18.29 per 100,000 population during the same period (EAPC = 0.55%; 95% CI = 0.46–0.64) (Table S5 and Fig. 2). In 2019, regions with middle SDI had the highest number of deaths (506,130), while regions with low SDI had the highest ASMR. Throughout the span of three decades, there was a decline in both the mortality rate and ASMR within low SDI regions (AAPC = -0.2; 95% CI = -0.22 to -0.19, EAPC = -0.27%; 95% CI = -0.31 to -0.22), while high-middle SDI regions experienced relatively stable rates. Conversely, high SDI regions demonstrated a significant increase in these rates (AAPC = 2.8; 95% CI = 2.76 to 2.84, EAPC = 1.21%; 95% CI = 1.09 to 1.32). Analyzing the five causes of CKD, all causes showed an increase in mortality rates and ASMR, with the most notable increase attributed to CKD caused by T2DM (AAPC = 2.21; 95% CI = 2.18 to 2.23), EAPC = 0.92%; 95% CI = 0.8 to 1.05) (Table S5 and Fig. 2).

Among geographical regions, South Asia had the highest number of deaths (285,342; 95%UI = 248,209 to 324,604), while Central Latin America demonstrated the highest ASMR (48.11 per 100,000 population; 95%UI = 42.52 to 54.16) (Table S5 and Fig. 1). The most significant increases in both mortality rate and ASMR were observed in Central Latin America (AAPC = 4.47; 95% CI = 4.32 to 4.56), EAPC = 1.44%; 95% CI = 1.31 to 1.58). Importantly, a notable decline in mortality rates has been observed in Eastern and Western Sub-Saharan Africa, as well as in Central Sub-Saharan Africa. The ASMR exhibited a downward trend across 8 regions, with the most significant reduction observed in High-income Asia Pacific (EAPC = -1.35%; 95% CI = -1.43 to -1.28), followed by Central Sub-Saharan Africa (EAPC = -0.64%; 95% CI = -0.68 to -0.61) and Eastern Sub-Saharan Africa (EAPC = -0.5%; 95% CI = -0.55 to -0.46) (Table S5 and Fig. 2).

Among the 204 countries and territories, India (222,922; 95%UI = 191,551 to 258,592), China 196,726; 95%UI = 168,241 to 224,684) and United States of America (2,287,706; 95%UI = 2,101,294 to 2,489,365) emerged as the three nations with the highest mortality rates. Notably, Estonia, Armenia, and El Salvador exhibited pronounced mortality trends. Mortality rates decreased in 39 countries and territories, with the most significant decrease observed in Afghanistan (AAPC = -2.67; 95% CI = -2.72 to -2.61). The highest ASMR was observed in Nicaragua (83.24 per 100,000 population; 95%UI = 69.57 to 96.75), followed by Micronesia (Federated States of) and Mauritius. Estonia (EAPC = 5.36%; 95% CI = 4.91 to 5.81), Armenia (EAPC = 4.65%; 95% CI = 4.33 to 4.97), and Latvia (EAPC = 4.52%; 95% CI = 4 to 5.05) exhibited the most substantial increase in ASMR among all countries. Notably, a downward trend in ASMR was observed in 78 countries, with Mongolia experiencing the largest decline (EAPC = -3.6%; 95% CI = -4.07 to -3.12) (Table S6). Between 1990 and 2019, there was an increase in the number of deaths across all countries except for Mongolia (-0.12%), where a slight decrease was observed. The United Arab Emirates experienced the most significant rise (593.20%) (Fig. 3).

### The disability-adjusted life years of CKD

In 2019, the number of DALYs worldwide was 41,538,592, an increase compared to 1990. Furthermore, an upward trend in the rate of DALYs was observed over the three-decade period (AAPC = 1.02; 95% CI = 0.99 to 1.04). The ASDR increased from 484.46 per 100,000 in 1990 to 514.86 per 100,000 in 2019, and the ASDR also showed an increasing trend (EAPC = 0.31%; 95% CI = 0.23 to 0.38) (Table S7 and Fig. 2). Among the five causes of CKD, CKD attributed to T2DM exhibited the highest increase in DALYs rate (AAPC = 1.8; 95% CI = 1.77 to 1.82). The ASDR for other and unspecified causes of CKD was recorded as the highest (146.24; 95%UI = 123.41 to 172.09), while the ASDR for CKD caused by T2DM demonstrated the most significant escalation (EAPC = 0.75%; 95% CI = 0.63 to 0.87). Conversely, there was minimal variation observed in CKD due to T1DM (EAPC = -0.08%; 95% CI = -0.18 to 0.02). The middle SDI region exhibited the highest number of DALYs, while the high SDI region had the second lowest number of DALYs among the five regions; however, it demonstrated the most pronounced growth trend (AAPC = 1.88; 95% CI = 1.86 to 1.9) (Table S7). The area with low SDI displayed the highest ASDR, whereas the area with high SDI showed the lowest ASDR. Meanwhile, the high SDI region was the area with the most obvious increase in ASDR, similar to the number of ASDR. The high-middle SDI region exhibited the lowest change in ASDR (EAPC = -0.36%; 95% CI = -0.46 to -0.27), while a similar negative growth was observed in the low SDI region (EAPC = -0.24%; 95% CI = -0.28 to -0.21).

Among the 21 GBD regions, South Asia had the highest number of DALYs (9,886,435; 95%UI = 8,796,773 to 11,052,833), and Central Latin America had the highest ASDR (1348.14 (1,203.58 to 1,521.61) (Table S7 and Fig. 1). The most significant increase in DALYs rate was seen in Central Latin America (AAPC = 3.58; 95% CI = 3.51 to 3.64), with three regions experiencing negative DALYs rate growth. The largest increase in ASDR was also seen in Central Latin America (EAPC = 2.3%; 95% CI = 2.01 to 2.59), with 13 regions showing negative ASDR growth (Table S7 and Fig. 2).

When 204 countries and regions were observed, the largest number of DALYs were in India (7,519,691; 95%UI = 6,550,348 to 8,500,578), China (5,831,843; 95%UI = 4,992,206 to 6,645,333), and the United States of America (2,287,706; 95%UI = 2,101,294 to 2,489,365) (Table S8). The largest increase in DALYs rate was seen in El Salvador (AAPC = 5.38; 95% CI = 5.08 to 5.62), followed by Armenia (AAPC = 5.03; 95% CI = 4.86 to 5.2) and Estonia (AAPC = 4.66; 95% CI = 4.2 to 5.03). Among them, 49 countries showed decreases in DALYs rate, with Ethiopia (AAPC = -2.36; 95% CI = -2.41 to -2.29), Afghanistan (AAPC = -2.1; 95% CI = -2.16 to -2.01) and Liberia (AAPC = -1.75; 95% CI = -1.84 to -1.68) showing the most significant reductions. The highest ASDR is in Micronesia (Federated States of Micronesia) (2,162.73 per 100,000 population; 95%UI = 1,584.61 to 2,761.55) and the lowest is in Finland (111.94 per 100,000 population; 95%UI = 97.61 to 129.02) (Table S8 and Fig. 1). A total of 77 out of 204 countries exhibited a declining trend, with Mongolia demonstrating the most substantial decrease (EAPC = -3.33%; 95% CI = -3.76 to -2.9), while El Salvador showcased the most significant increase (EAPC = 3.97%; 95% CI = 3.32 to 4.61) (Table S8 and Fig. 2). When examining the progression of DALYs from 1990 to 2019, the United Arab Emirates (632.10%) and Qatar (508.32%) exhibited the most substantial growth rates. Conversely, two countries experienced negative growth: Slovakia with a marginal decline (0.22%), and Poland with a significant decrease (-16.44%) (Fig. 3).

### Age and gender-based analysis of CKD

**Fig. 4:**
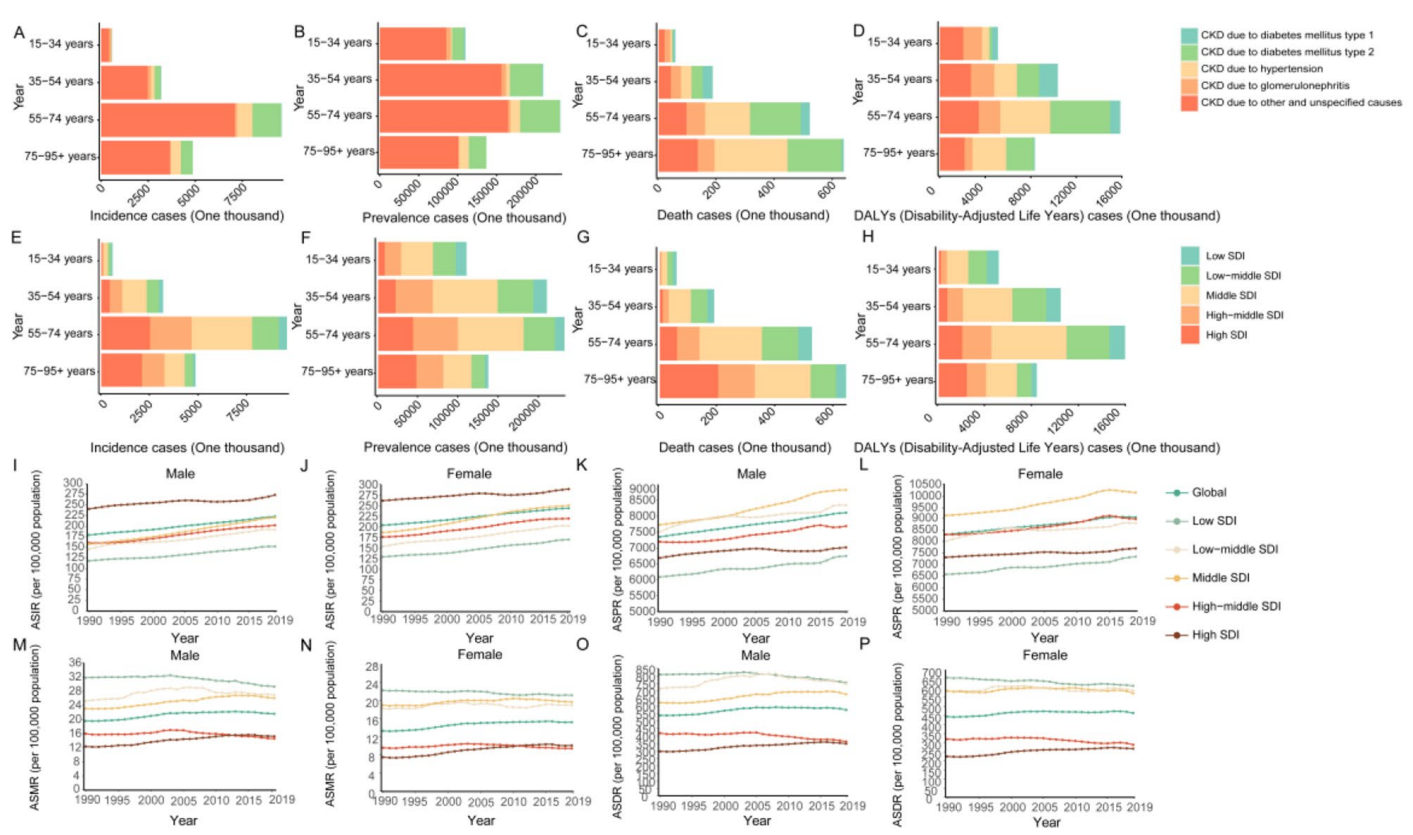
The incidence (**A**), prevalence (**B**), mortality (**C**) and disability-adjusted life years cases (**D**) of CKD caused by different pathogenic factors in various age groups (15–34 years, 35–54 years, 55–74 years and 75–95 + years). The incidence (**E**), prevalence (**F**), mortality (**G**) and disability-adjusted life years cases (**H**) of CKD in five SDI quintiles (Low SDI, Low-middle SDI, Middle SDI, High-middle SDI and High SDI). Age-standardized incidence rates, age-standardized prevalence rates, age-standardized mortality rates, and age-standardized disability adjusted life years rates were calculated by region and gender (I-P)

To assess disparities in CKD by age, we divided the population into four age groups: 15–34 years, 35–54 years, 55–74 years, and 75–95 + years for detailed analysis. Our findings show that the majority of CKD cases, both in terms of incidence and DALYs, occurred predominantly in individuals aged 55–74 years (Fig. 4A, D). Additionally, mortality from CKD primarily affected those over 55, with other and unspecified causes leading the incidence, followed by type 2 diabetes mellitus (T2DM). For prevalence, the patterns mirrored those of incidence, with type 1 diabetes mellitus (T1DM), hypertension, and glomerulonephritis forming a smaller share (Fig. 4B). In terms of mortality causes, no significant differences were observed between the 15–34 and 35–54 years age groups, but the impact of hypertension and T2DM grew markedly among those aged 55–75 years and those over 75 (Fig. 4C). In younger cohorts (15–34 and 35–54 years), a substantial portion of CKD-related DALYs were attributed to other and unspecified causes. The main causes of CKD in older age groups (55–74 and 75–95 + years) were T2DM and hypertension (Fig. 4D).

Further analysis across different Socio-demographic Index (SDI) quintiles indicated that the 55–74 age group had the highest case numbers in all quintiles, with the middle SDI quintile showing the highest counts. Generally, the 35–54 and 55–74 years age groups had a higher prevalence in the middle SDI ranges, whereas lower counts were noted in the low SDI ranges. Mortality rates increased with age, peaking in the 75–95 + age group. The pattern of deaths across SDI quintiles revealed a gradual increase with age, with the highest numbers in the medium, low-medium, and low SDI quintiles occurring in the 55–74 age group (Fig. 4E-H).

Gender-based analyses (Fig. 4I-P) indicated minimal disparities across SDI quintiles. Globally and across SDI quintiles, females showed slightly higher age-standardized incidence rates (ASIR) and prevalence rates (ASPR) compared to males; conversely, males had higher age-standardized mortality rates (ASMR) and disability-adjusted life years rates (ASDR) than females. The highest ASIR for both genders were in the high SDI quintile, surpassing global levels. For ASPR, males in the middle and low-middle SDI quintiles showed higher rates compared to global levels, whereas for females, rates in the low-middle and high-middle SDI quintiles were similar to global rates. The pattern for ASMR and ASDR was consistent, showing the highest rates in the low SDI quintile, with males in the low-middle, middle, and high SDI quintiles displaying the lowest rates. Females in the high SDI quintile exhibited the lowest ASMR and ASDR, with their rates in the low-middle and middle SDI quintiles being comparable. Meanwhile, Fig. S1 illustrated that ASIR and ASPR are higher in males than in females, while ASMR and ASDR are the opposite.

### Drivers of CKD epidemiology – ageing, population growth, and epidemiological change

To investigate the impact of ageing, epidemiological changes, and population growth on the epidemiology of CKD from 1990 to 2019, we developed a decomposition analysis of raw incidence, prevalence, mortality, and DALYs based on aging, epidemiologic changes (standardized morbidity and mortality rates) and population growth.

**Fig. 5:**
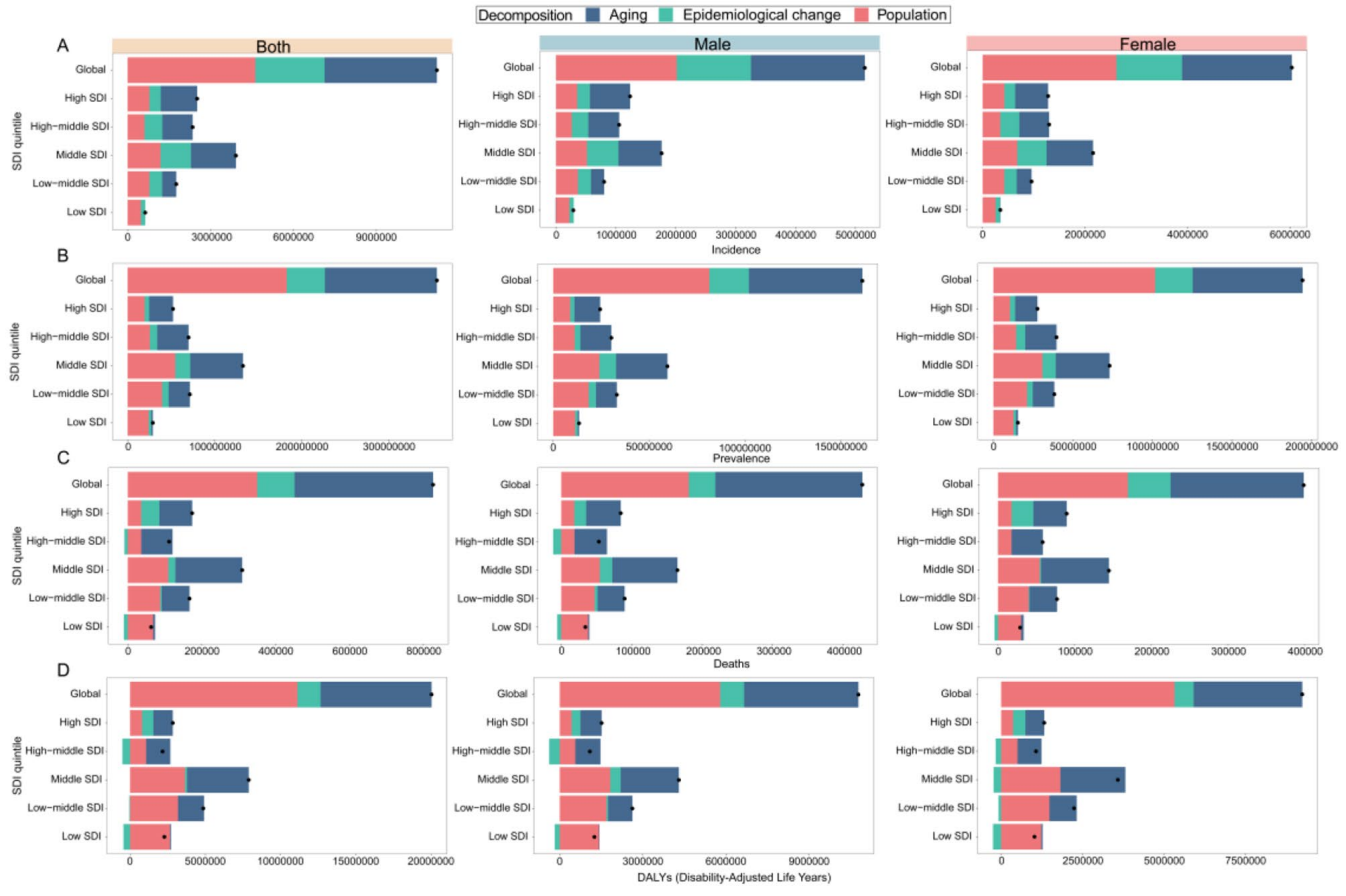
Global changes in the incidence (**A**), prevalence (**B**), mortality (**C**) and DALYs (**D**) of CKD in relation to aging, epidemiologic change, population growth and SDI quintiles from 1990 to 2019. The black dots represent the overall change values of the contributions of all 3 components. For each component, the magnitude of a positive value indicates a corresponding increase in CKD caused by that component. The magnitude of the negative values indicates that the corresponding reduction in CKD is attributed to the relevant component

Globally, the incidence, prevalence, mortality, and DALYs of CKD are on the rise, with increasing impact from aging, epidemiologic changes, and population growth. In terms of the incidence rate, the increase was mainly affected by population growth (41.31%), followed by aging (36.31%). From 1990 to 2019, aging exerted the most substantial impact on regions with High SDI at a rate of 52.36%. This impact gradually diminished in regions with high-medium-, medium-, and low-medium SDI regions, while it was almost negligible in low SDI regions (0.16%). The incidence rate experienced a more pronounced increase in the middle SDI compared to the other four SDI regions, primarily driven by the burden of aging which accounted for 41.56% of this change. Conversely, the low SDI region exhibited minimal changes in incidence rates mainly due to population growth (75.19%) (Fig. 5A; Table 1).

Overall, the global prevalence increase was primarily driven by population (51.44%), while epidemiologic changes had a relatively minor impact (12.33%). Among the SDI quintiles, the middle SDI quintile exhibited the most significant rise. Similar to the incidence, aging exhibited the greatest influence on the high SDI quintile (52.61%), followed by decreasing impacts in descending order for other regions, with minimal effect observed in the low SDI quintile (6.60%). Most notably, in contrast to the impact of aging on SDI quintiles, the effect of aging exhibited an ascending trend from high SDI quintile to low SDI quintile, namely 37.45%, 36.59%, 41.31%, 55.44%, and 83.43% respectively. The influence of epidemiologic changes on CKD prevalence demonstrated relatively stable fluctuations across the five SDI quintiles, ranging from 9 to 13% (Fig. 5B; Table 1).

During the 30-year period, the mortality of CKD was primarily influenced by aging (45.40%) and population growth (42.39%), while epidemiologic changes had a limited impact on CKD mortality (12.21%). Notably, the middle SDI quintile exhibited the largest increase in CKD mortality. The impact of epidemiologic changes on CKD mortality decreased in high-medium SDI quintile and low SDI quintile. In the high-medium SDI quintile, aging emerged as the most significant factor contributing to CKD mortality (accounting for 75.84%), exerting a greater influence compared to the other four SDI quintiles. In the low SDI quintile, in contrast to the high-medium SDI region, the impact of aging on the low SDI quintile was minimal (8.45%), while the influence of population growth was significantly pronounced (108.60%) (Fig. 5C; Table 1).

The global DALYs rate of CKD was primarily influenced by population growth (55.52%), followed by population aging (36.82%), with epidemiologic changes having the least impact (7.66%). Notably, the influence of epidemiologic changes on the DALYs rate of CKD was diminished with high-medium SDI, low-medium SDI, and low SDI quintile. Similar to the patterns observed in CKD mortality, the rise in DALYs rates of CKD was most pronounced in middle SDI quintile. Aging had a significant impact on high-middle SDI quintile (74.57%) but a minimal effect on those with low SDI quintile (3.04%). The influence of population growth was most substantial in low SDI quintile (116.35%) and less significant in those with high SDI quintile (27.91%) (Fig. 5D; Table 1).

**Table 1:** Age-standardized rate for CKD incidence, prevalence, mortality and DALYs, and percentage change from 1990 globally and by SDI quintile

Location	Overall difference	Incidence changes due to population-level determinants*			Overall difference	Prevalence changes due to population-level determinants*		
		Aging	Epidemiologic changes	Population		Aging	Epidemiologic changes	Population
Global	11,190,575	4,062,899 (36.31%)	2,504,662 (22.38%)	4,623,015 (41.31%)	355,628,727	128,836,443 (36.23%)	43,839,120 (12.33%)	182,953,164 (51.44%)
High SDI	2,515,612	1,317,277 (52.36%)	409,355 (16.27%)	788,980 (31.36%)	52,220,767	27,475,062 (52.61%)	5,187,526 (9.93%)	19,558,179 (37.45%)
High-middle SDI	2,352,164	1,096,555 (46.62%)	643,558 (27.36%)	612,051 (26.02%)	70,047,763	36,028,864 (51.43%)	8,385,858 (11.97%)	25,633,042 (36.59%)
Middle SDI	3,919,423	1,629,060 (41.56%)	1,099,990 (28.07%)	1,190,373 (30.37%)	132,640,959	60,632,059 (45.71%)	17,215,583 (12.98%)	54,793,318 (41.31%)
Low-middle SDI	1,759,392	510,652 (29.02%)	459,582 (26.12%)	789,159 (44.85%)	71,544,023	24,433,408 (34.15%)	7,447,516 (10.41%)	39,663,100 (55.44%)
Low SDI	636,710	1027 (0.16%)	156,960 (24.65%)	478,723 (75.19%)	28,958,173	1,911,574 (6.60%)	2,886,189 (9.97%)	24,160,411 (83.43%)
Location	Overall difference	Mortality changes due to population-level determinants*			Overall difference	DALYs changes due to population-level determinants*		
		Aging	Epidemiologic changes	Population		Aging	Epidemiologic changes	Population
Global	825,925	374,932 (45.40%)	100,884 (12.21%)	350,109 (42.39%)	20,034,022	7,377,149 (36.82%)	1,534,475 (7.66%)	11,122,398 (55.52%)
High SDI	174,118	88,796 (51.00%)	49,042 (28.17%)	36,280 (20.84%)	2,836,831	1,295,203 (45.66%)	749,976 (26.44%)	791,652 (27.91%)
High-middle SDI	111,660	84,682 (75.84%)	-9344 (-8.37%)	36,322 (32.53%)	2,158,988	1,609,950 (74.57%)	-514,477 (-23.83%)	1,063,516 (49.26%)
Middle SDI	309,540	180,807 (58.41%)	19,314 (6.24%)	109,419 (35.35%)	7,884,029	4,096,712 (51.96%)	139,623 (1.77%)	3,647,694 (46.27%)
Low-middle SDI	166,977	75,344 (45.12%)	3811 (2.28%)	87,822 (52.60%)	4,863,576	1,733,526 (35.64%)	-51,140 (-1.05%)	3,181,191 (65.41%)
Low SDI	63,054	5331 (8.45%)	-10,756 (-17.06%)	68,479 (108.60%)	2,274,783	69,263 (3.04%)	-441,102 (-19.39%)	2,646,622 (116.35%)

DALYs – disability-adjusted life years, SDI – Socio-demographic Index

^*^Percentages contribute to the total changes

### HAQ and SDI

**Fig. 6:**
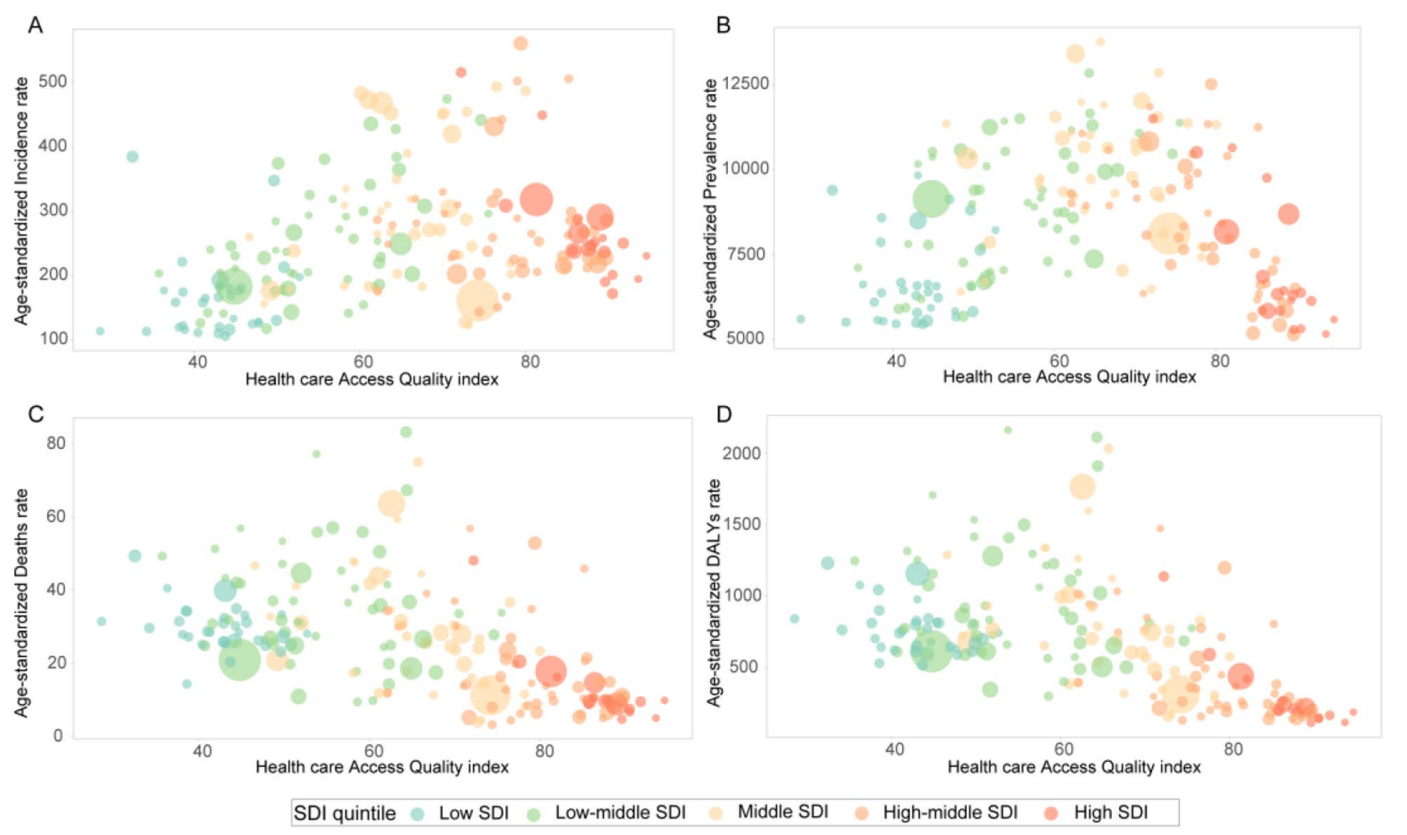
Association between age-standardized incidence (**A**), prevalence (**B**), mortality (**C**) and DALYs (**D**) rate and HAQ index. Each circle represents a country. Circles are colored according to SDI quintile. Circle size corresponds to population number

To investigate the correlation between HAQ and CKD burden, we conducted an analysis on the association between age-standardized rates and HAQ. The findings revealed that countries with moderate HAQ exhibited higher ASIR and ASPR. Conversely, countries with low HAQ demonstrated higher ASMR and ASDR, while those with high HAQ displayed lower ASMR and ASDR. In conclusion, countries with limited coping capacity bear a significant burden of CKD (Fig. 6).

**Fig. 7:**
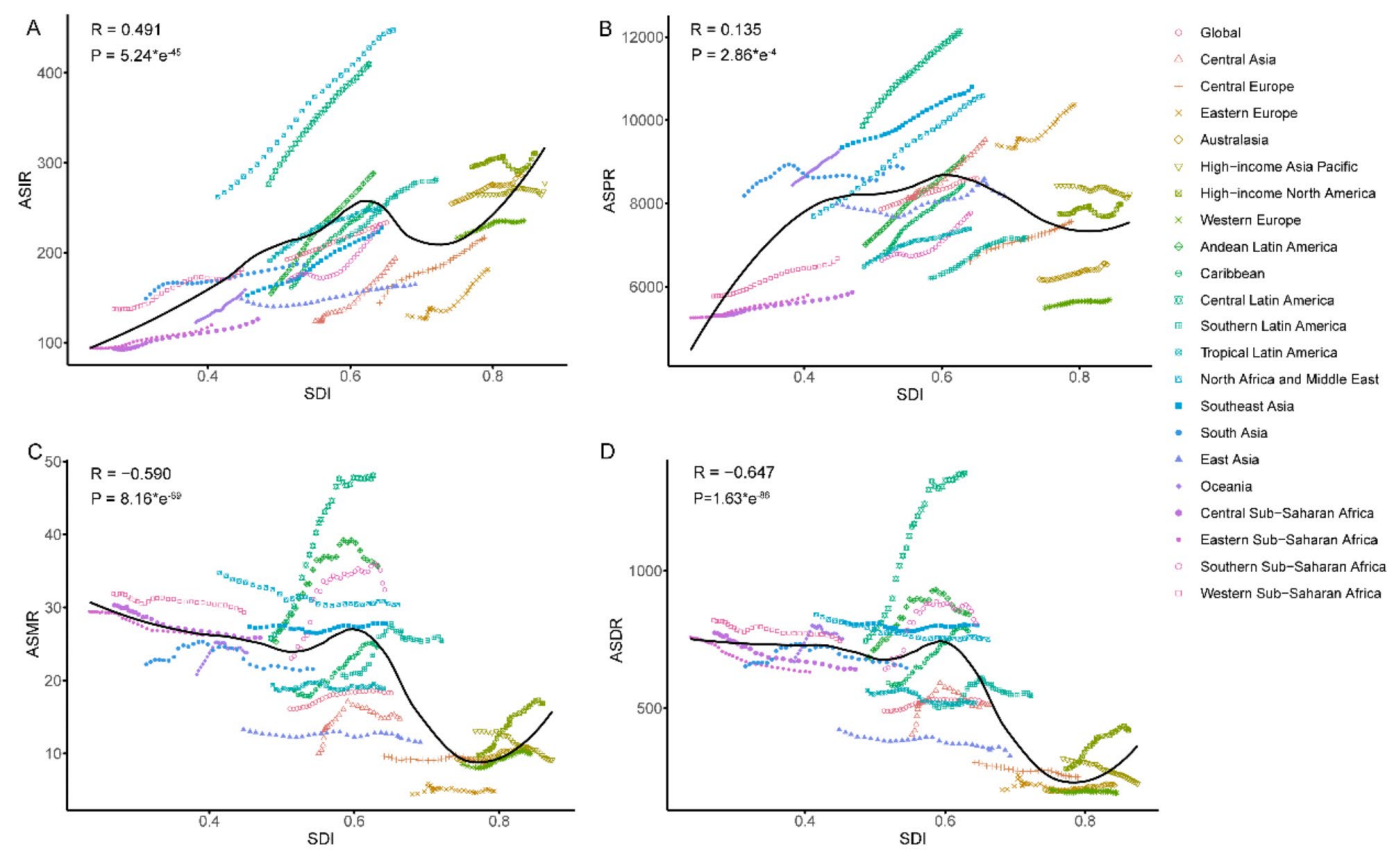
Age-standardized incidence rates (**A**), age-standardized prevalence rates (**B**), age-standardized mortality rates (**C**) and age-standardized disability-adjusted life years rates (**D**) for CKD for 21 GBD regions by Socio-demographic Index, 1990–2019

Overall, there were disparities observed in the associations of age-standardized rates and SDI on a global as well as within the GBD regions during the period 1990–2019. A positive correlation was found between ASIR and SDI, while a negative correlation was identified between ASMR, ASDR, and SDI. Additionally, a weak correlation was observed between ASPR and SDI (Fig. 6B). Observing the correlation between ASIR and SDI, the median SDI was significantly different from the expected value. At a global scale, North Africa, the Middle East and Central Latin America exhibited significantly higher values than anticipated, whereas Central and East Asia along with Eastern Europe demonstrated significantly lower values than expected (Fig. 6A). The overall correlation trend between ASMR and ASDR and SDI was consistent, while the correlations with SDI varied significantly across different regions (Fig. 6C-D). Notably, there was a clear downward trend in the correlation between certain regions of Low SDI and low-middle SDI with SDI. Conversely, the remaining regions of low-middle SDI exhibited an upward trend, whereas the middle SDI experienced a significant decline. Lastly, both high-middle and high SDI showed an upward trend. The regions of South Asia, Oceania, Andean Latin America, Central Latin America, Sub-Saharan Africa, the Caribbean, Central Asia, southern Latin America, and high-income North America exhibited a positive correlation with SDI, while the remaining regions demonstrated either a relatively stable or negative correlation. The correlation between age-standardized rate and SDI in different countries and regions is shown in the Figs. 7 and S2. ASIR and ASPR are positively correlated with SDI, while ASMR and ASDR are negatively correlated with SDI.

### CKD burden attributed to each risk factor by SDI

**Fig. 8:**
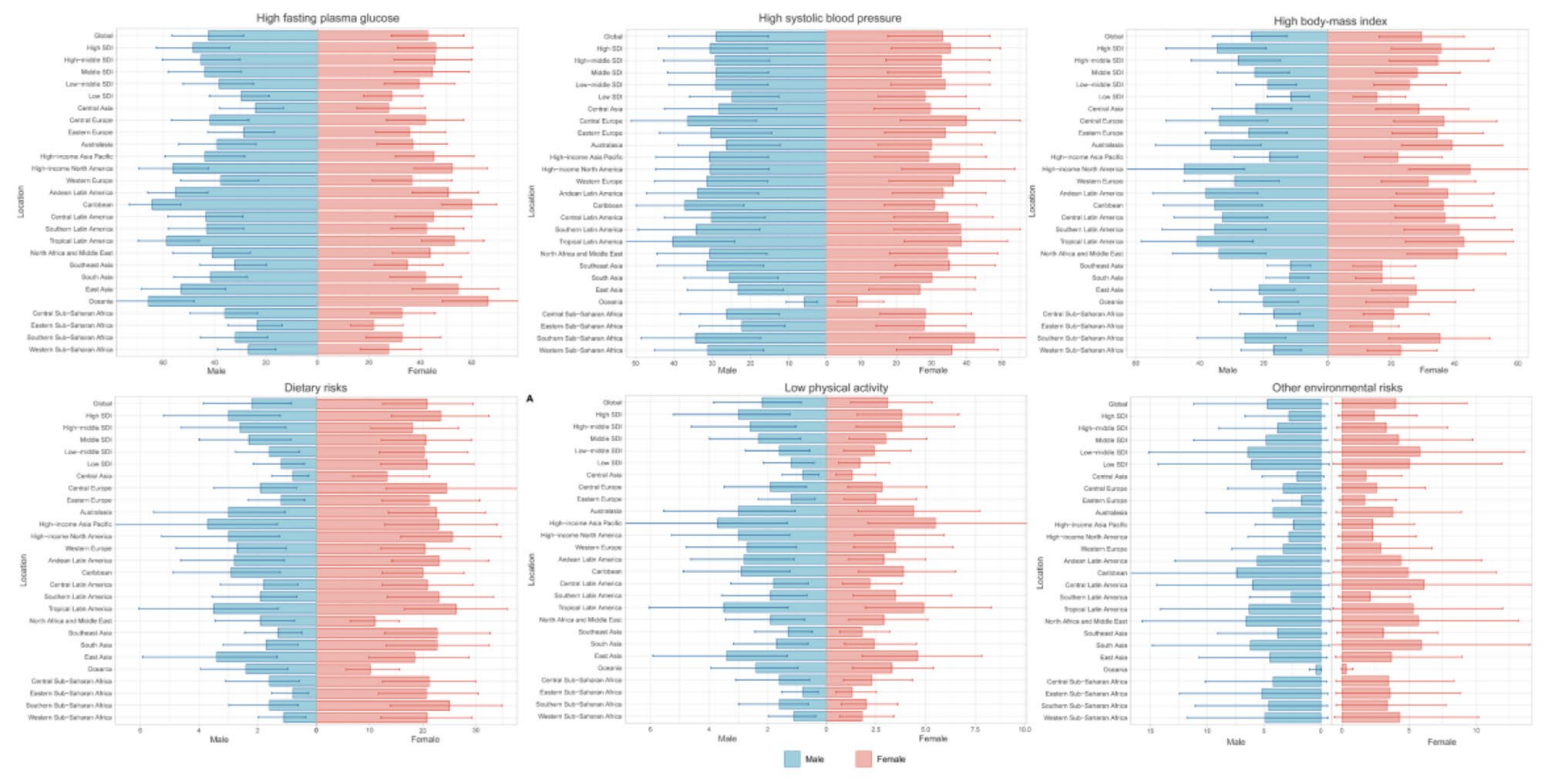
The PAF of these six risk factors for CKD deaths in 2019

In Fig. 8, it is evident that high fasting plasma glucose and high systolic blood pressure remain the leading contributors to CKD-related deaths. The PAF decreases more pronouncedly from high to low SDI regions. High BMI, dietary risks, and low physical activity have a greater impact in higher SDI regions, whereas environmental risks exert a more significant influence in lower SDI areas.

**Fig. 9:**
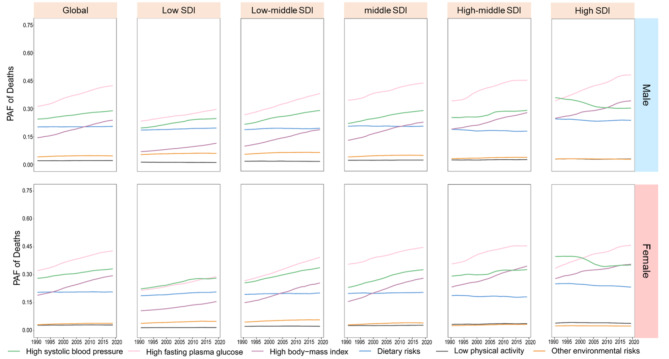
Contributions of 6 risk factors to the PAF of deaths due to CKD by different SDI quintiles and sex from 1990 to 2019

Over the past 30 years, high systolic blood pressure has shown a declining trend in high SDI regions (Fig. 9), while it has been on the rise in other SDI categories. Despite the downward trend in high SDI regions, the impact of hypertension on CKD-related deaths remains substantial. Furthermore, apart from high systolic blood pressure, the PAF for all other factors has been consistently rising over the past three decades.

## Discussion

We conducted a detailed analysis of the incidence, prevalence, mortality, and disability-adjusted life years (DALYs) associated with chronic kidney disease (CKD) in adults aged 20 to over 95 years, accounting for age and gender as critical factors. Our results highlight significant upward trends in all four metrics, illustrating a progressive increase in the burden of CKD over the past three decades. The incidence of CKD in 2019 marked a substantial rise of 144% from 1990, affecting nearly 19 million individuals. Similarly, the prevalence of CKD escalated by 104%, encompassing over 697 million people globally. Tragically, the disease led to approximately 1.4 million deaths, a distressing increase of 137%. Moreover, more than 41 million healthy life years were lost due to CKD, representing an alarming growth of 93% in DALYs.

The decomposition analysis indicated that the burden of CKD was primarily driven by demographic factors, specifically population growth and aging, with epidemiological changes having a lesser impact. Over the past three decades, countries within the middle SDI quintiles consistently faced the highest disease burden, as measured by incidence, prevalence, mortality, and DALYs. This trend may be attributed to the larger population size within these middle SDI quintiles, which renders them more vulnerable to the effects of demographic shifts and aging.

CKD ranked as the 18th leading cause of mortality in 1990, but advanced to the 11th position by 2019 [37]. Nevertheless, apart from an increase in the age-standardized mortality rate (ASMR) for CKD attributed to glomerulonephritis, the observed decline in both CKD cases and CKD-related deaths across all causes since 2016 highlights global progress in CKD management. However, the management of glomerulonephritis requires further improvement. Interestingly, the incidence and prevalence of CKD were found to be higher in females, while mortality and DALYs were higher in males, suggesting a potentially accelerated progression towards ESRD among males. This discrepancy may be linked to less favorable lifestyle choices in males, as well as the potential protective effects of estrogen or the detrimental impacts of testosterone.

From 1990 to 2019, there was a marginal global decline in disability-adjusted life years (DALYs) for all diseases, with figures decreasing from 2,593,478,052.39 (95% CI = 23,065,777.45 to 2,440,466,782.64) in 1990 to 2,538,020,070.65 (95% CI = 2,285,262,550.96 to 2,810,205,654.82) in 2019. Additionally, the age-standardized disability-adjusted life years rate (ASDR) showed a significant decrease from 50,059.93 per 100,000 in 1990 to 32,856.98 per 100,000 in 2019, reflecting substantial improvements in global health. In contrast, DALYs and ASDR for CKD showed a significant increase, from 21,504,570.60 to 41,538,592.34 and from 484.46 to 514.86, respectively. This trend positions CKD as the twelfth leading risk factor, underscoring the growing disease burden of CKD over the past three decades and its rising prominence as a major contributor to the global burden of disease. Simultaneously, there is an ongoing shift in the global disease burden from communicable to non-communicable diseases, with CKD comprising an increasing share of the non-communicable disease burden (from 1.9 to 2.6%). This transition further highlights the significant impact CKD continues to impose on global health [38].

The impact of hypertension on CKD primarily manifests in the damage it inflicts on target organs of patients, as well as its effects on cardiovascular outcomes. This may be attributed to the association of low estimated glomerular filtration rate (eGFR) and albuminuria with an increased risk of cardiovascular mortality [39]. Over the past 30 years, except for high SDI regions, the PAF has shown an increasing trend in other regions. This may be attributed to high SDI areas having greater funding and better educational resources, which enhance patients’ understanding of their conditions, allowing for more effective diagnosis and treatment. According to the KDIGO 2021 Clinical Practice Guideline for the Management of Blood Pressure in Chronic Kidney Disease, it is recommended to maintain systolic blood pressure below 120 mmHg for optimal outcomes. In low SDI regions, such as Africa, a study indicated that only 51.4% of hypertensive patients were aware of their diagnosis, and 51.8% had used antihypertensive medication within the previous two weeks [40]. This region still requires national efforts to strengthen healthcare measures for better management of hypertensive patients.

Hypertension and hyperglycemia are established risk factors for diabetes, and diabetic kidney disease is a major contributor to the global burden of disease [41]. Over the past 30 years, the has shown an increasing trend across all regions, making it the highest contributing factor to mortality from chronic kidney disease (CKD). This indicates that, regardless of economic status, control of hyperglycemia is poor in various regions, representing the most significant risk factor among the six identified for CKD and posing a substantial challenge for its management. We recommend an individualized HbA1c target range of < 6.5% to < 8.0% for diabetic patients with CKD who are not receiving dialysis, along with a low-salt diet and moderate-intensity physical activity [42].

Recent studies indicate that diet-related risks for CKD primarily stem from protein-associated dietary intake. High-protein diets may lead to increased urinary albumin excretion and an initial rise in GFR, followed by a subsequent decline. Additionally, there is growing evidence that high-protein diets may be associated with several metabolic complications that could be detrimental to kidney health [43]. Over the past 30 years, the PAF related to dietary risks has remained relatively stable, but it still constitutes a significant proportion of mortality risk. The 2024 KDIGO guidelines recommend that a whole-food, plant-based diet low in animal-based and ultra-processed foods may help mitigate the progression of CKD, while also alleviating cardiovascular factors such as hypertension and diabetes that contribute to CKD [44].

High BMI and low physical activity are contributing factors to obesity, and the relationship between obesity and CKD has been increasingly explored in recent years. An article published in the Lancet examined the impact of obesity on global health, noting that affluent countries bear a higher burden of obesity compared to low- and middle-income countries. Countries transitioning from low to high income have experienced rapid urbanization and a shift towards motorized transportation, resulting in decreased physical activity and increased obesity prevalence [45]. This aligns with our findings, which indicate that as the SDI decreases, the PAF associated with BMI and low physical activity also declines, highlighting the significant impact of these factors in high-income regions. To address these issues, a global collaborative effort is necessary to develop reasonable measures and enhance governance benchmarks. Furthermore, high SDI regions need to refine relevant policies and strengthen urban governance to drive transformative change.

An increasing body of evidence indicates that environmental air pollution, particularly particulate matter (PM), nitrogen dioxide (NO_2_), and nitrogen oxides (NO), are major risk factors for the development of hypertension, diabetes, and CKD [46, 47]. It is evident that the PAF associated with environmental pollution gradually decreases with an increase in the SDI, likely due to better economic conditions in wealthier countries, which generally experience lower levels of environmental pollution and more effective environmental protection measures. A study based on data from the UK Biobank demonstrated that PM_2.5_, PM_10_, NO_2_, and NO_x_ are associated with an increased risk of transitioning from health to newly diagnosed hypertension, diabetes, and CKD during their dynamic development [47]. However, recent guidelines do not include measures related to the prevention of environmental pollution. Based on our research findings, we advocate for the incorporation of environmental pollution control as a primary prevention strategy for CKD, highlighting the urgent need for relevant authorities to establish corresponding laws and regulations.

The disease burden of CKD shows distinct regional and developmental disparities. For the SDI, the ASMR and ASDR for high and high-middle SDI countries are relatively lower compared to the other three SDI categories, particularly in the high-middle SDI group, where the EAPC in ASMR and ASDR has remained fairly stable. Observing data across 204 countries reveals that the United States, Germany, Canada, the United Kingdom, Turkey, Iran, and Japan all exhibit high ASIR and low ASDR, collectively indicating that the management of CKD is associated with the economic development status of these nations. Our findings reveal a concentrated incidence and prevalence of CKD in North Africa and the Middle East, with these regions exhibiting higher numbers compared to others. This discrepancy could be attributed to the unhealthy dietary habits and lifestyles prevalent among populations in these areas. Such factors likely contribute to the elevated rates of CKD, underscoring the need for targeted health interventions to address these modifiable risk factors [48]. Furthermore, studies have demonstrated an association between non-communicable diseases and education level, which is comparatively low in this particular region, thereby impacting disease awareness. The level of medical and health care is relatively inadequate, and there exists a lack of timely adjustment in public health policies [49].

The overall levels of ASMR and ASDR for CKD are notably high in Africa, while certain countries in Asia, North America, and South America also display elevated levels. Europe, in contrast, shows the lowest levels. Additionally, variations in ASIR, ASPR, ASMR, and ASDR for CKD are observed across different countries globally. Particularly in Sub-Saharan Africa, despite lower levels of ASIR and ASPR, the region experiences higher rates of ASMR and ASDR, highlighting the critical role of renal replacement therapy. Evidence suggests that access to renal replacement therapy in both high-income and low-income areas is significantly greater—approximately 200 times—than in Sub-Saharan Africa. A study conducted in sub-Saharan Africa indicates that among adults with newly diagnosed renal failure requiring dialysis, only 10% are able to continue dialysis for three months or longer, and just 1% can sustain treatment for 12 months or more. Furthermore, it is estimated that 96% of adults known or presumed to have died did so due to an inability to afford medical expenses [50]. This disparity underscores the urgent need for enhanced access to renal health services and interventions in regions most affected by CKD to mitigate its impact and improve patient outcomes [51]. Consequently, a majority of patients in these regions are unable to initiate treatment due to prohibitive costs or are compelled to discontinue treatment prematurely, which significantly compromises life expectancy. The analysis of age-standardized rates in conjunction with the SDI and the HAQ index further illustrates this predicament. Typically, lower SDI quintiles and HAQ scores are associated with lower ASIR and ASPR but higher ASMR and ASDR for CKD. This pattern underscores that the burden of CKD is disproportionately concentrated in economically less developed regions, highlighting a critical need for enhanced healthcare access and affordability in these areas to alleviate the impact of CKD.

Several limitations of this study warrant mention. First, it is important to note that our investigation primarily focuses on a macro assessment of global, regional, and national epidemiological trends of CKD in adults. Consequently, the analysis does not encompass CKD in minors, thereby limiting its ability to provide a comprehensive overview of the entire spectrum of this disease. Second, despite the increased granularity of statistics in the GBD database, there are still certain countries or regions with insufficient data, which inevitably introduces potential bias into the datasets. Third, we did not perform an in-depth analysis of country-specific data, suggesting that caution should be exercised when extrapolating the findings to countries with low HAQ scores. Furthermore, in the decomposition analysis method, it is challenging to isolate the impact of population aging while keeping the population proportion constant, and vice versa. More crucially, the data on diabetes-induced nephropathy is primarily based on information from the Geisinger Health System (a renowned health management organization in Pennsylvania, USA), which has been extended to other countries’ contexts. Our study also highlights several notable strengths. It leverages data from the comprehensive 1990–2019 GBD database, which represents the most extensive compilation of global health information available to date. Furthermore, we employed diverse models and conducted decomposition analyses to facilitate cross-country and regional comparisons, enhancing our understanding of the epidemiology of CKD across different settings.

In summary, our epidemiological analysis of CKD patients offers valuable insights for managing this condition. As the burden of CKD is primarily concentrated in economically disadvantaged regions, a study assessing the readiness and capacity for kidney care across various countries revealed that only 21 (18%) and 9 (8%) countries reported consistent availability of serum creatinine and eGFR and proteinuria measurements for CKD monitoring in primary healthcare settings. Additionally, public funding and free care for hemodialysis, peritoneal dialysis, and transplant services are provided in 50 (42%), 48 (51%), and 46 (49%) countries, respectively [52]. Therefore, enhancing CKD monitoring and patient care can significantly improve disease management. Given the limited dialysis and transplant services in low-income countries, early intervention to manage patient symptoms can help delay disease progression.

## Conclusions

In conclusion, compared to 1990, there has been a significant increase in the incidence, prevalence, mortality, and DALYs attributed to CKD. This rise in CKD-related indicators can primarily be attributed to population growth and aging. Furthermore, variations were observed across different age groups and genders; female exhibited higher ASIR and ASPR, while male had higher ASMR and ASDR. Moreover, the disproportionate burden of CKD on regions with underdeveloped economies and suboptimal health system performance underscores the need for effective public health strategies to address global disparities in CKD.

## Electronic Supplementary Material

Below is the link to the electronic supplementary material.

Supplementary Material 1

Supplementary Material 2

## Data Availability

The data utilized in this study are fully accessible from the GBD database (https://ghdx.healthdata.org/gbd-2019). Data generated during the research process can be obtained through supplementary files or by contacting the corresponding author.

## Abbreviations

CKD: Chronic kidney disease
DALYs: Disability-adjusted life years
HAQ: Healthcare Quality and Access
SDI: Socio-demographic Index
ESRD: End-stage renal disease
COPD: Chronic obstructive pulmonary disease
GBD: Global Burden of Disease
ASR: EAPC Age-standardized rate of estimated annual percentage change
ASIR: Age-standardized incidence rates
ASPR: Age-standardized prevalence rates
ASMR: Age-standardized mortality rates
ASDR: Age-standardized disability-adjusted life years rates
AAPCs: Average annual percent changes
CI: Confidence interval
T1DM: Type 1 diabetes mellitus
T2DM: Type 2 diabetes mellitus
EAPC: Estimated annual percent change
PAF: Population attributable fraction
BMI: High body mass index
