# Supplementary material for: Epidemiological shifts in chronic kidney disease: a 30-year global and regional assessment
Source: BMC Public Health. 2024 Dec 18;24:3519. doi: 10.1186/s12889-024-21065-9 (PMC11657796; doi:10.1186/s12889-024-21065-9)
Supplement: Supplementary file 2 — Supplementary Material 2 [file 12889_2024_21065_MOESM2_ESM.pdf]

Supplementary Material

Epidemiological Shifts in Chronic Kidney Disease: A 30-Year Global and Regional Assessment

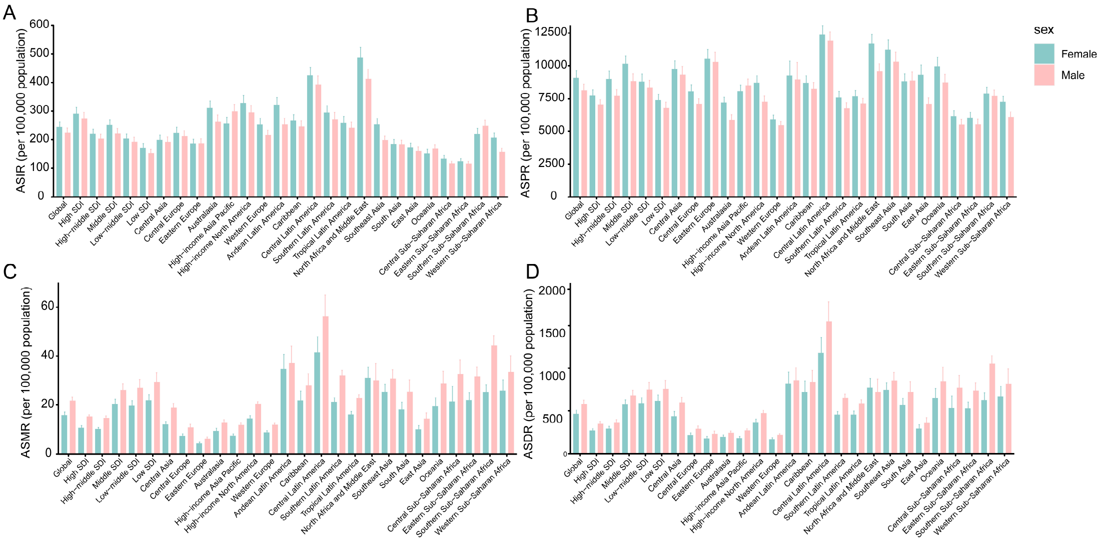

Figure S1 Age-standardized incidence rates (A), age-standardized prevalence rates (B), age-standardized mortality rates (C) and age-standardized disability-adjusted life years rates (D) for CKD for globally and 27 GBD regions by gender.

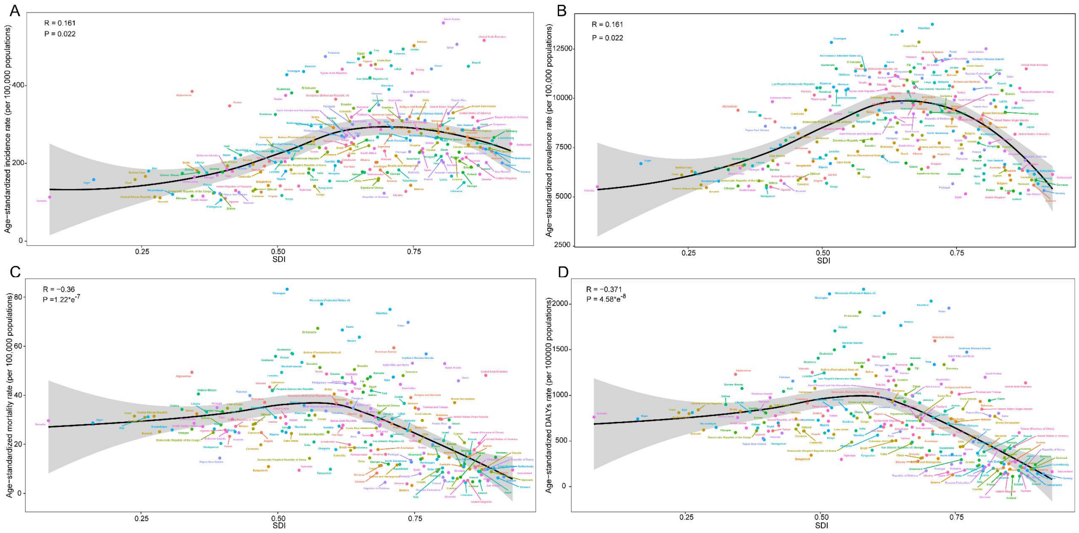

Figure S2 Age- standardized incidence rates (A), age-standardized prevalence rates (B), age-standardized mortality rates (C) and age-standardized disability-adjusted life years rates (D) for CKD for 195 countries and territories by Socio-demographic Index, 1990–2019.
